# Changes in Scottish suicide rates during the Second World War

**DOI:** 10.1186/1471-2458-6-167

**Published:** 2006-06-23

**Authors:** Rob Henderson, Cameron Stark, Roger W Humphry, Sivasubramaniam Selvaraj

**Affiliations:** 1 NHS Highland, Department of Public Health, Assynt House, Beechwood Park, Inverness, IV2 3HG, Scotland, UK; 2 Centre for Rural Health, University of Aberdeen, The Green House, Beechwood Business Park North, Inverness, IV2 3ED, Scotland, UK; 3 Epidemiological Research Unit, Scottish Agricultural College, Drummond Hill, Inverness, IV2 4JZ, Scotland, UK

## Abstract

**Background:**

It is believed that total reported suicide rates tend to decrease during wartime. However, analysis of suicide rates during recent conflicts suggests a more complex picture, with increases in some age groups and changes in method choice. As few age and gender specific analyses of more distant conflicts have been conducted, it is not clear if these findings reflect a change in the epidemiology of suicide in wartime. Therefore, we examined suicide rates in Scotland before, during and after the Second World War to see if similar features were present.

**Methods:**

Data on deaths in Scotland recorded as suicide during the period 1931 – 1952, and population estimates for each of these years, were obtained from the General Register Office for Scotland. Using computer spreadsheets, suicide rates by gender, age and method were calculated. Forward stepwise logistic regression was used to assess the effect of gender, war and year on suicide rates using SAS V8.2.

**Results:**

The all-age suicide rate among both men and women declined during the period studied. However, when this long-term decline is taken into account, the likelihood of suicide during the Second World War was higher than during both the pre-War and post-War periods. Suicide rates among men aged 15–24 years rose during the Second World War, peaking at 148 per million (41 deaths) during 1942 before declining to 39 per million (10 deaths) by 1945, while the rate among men aged 25–34 years reached 199 per million (43 deaths) during 1943 before falling to 66 per million (23 deaths) by 1946. This was accompanied by an increase in male suicides attributable to firearms and explosives during the War years which decreased following its conclusion.

**Conclusion:**

All age male and female suicide rates decreased in Scotland during World War II. However, once the general background decrease in suicide rates over the whole period is accounted for, the likelihood of suicide among the entire Scottish population during the Second World War was elevated. The overall decrease in suicide rates concealed large increases in younger male age groups during the War years, and an increase in male suicides recorded as due to the use of firearms. We conclude that the effects of war on younger people, reported in recent conflicts in Central Europe, were also seen in Scotland during the Second World War. The results support the findings of studies of recent conflicts which have found a heterogeneous picture with respect to age specific suicide rates during wartime.

## Background

Suicide rates are thought to fall during wartime. Durkheim [1] suggested this was due to increased social cohesion, while others have suggested that competing outcomes may be important, with individuals who might otherwise have died by suicide being more likely to die of other causes [2]. Neeleman [3] cites this as an example of contextual effect modification, with suicide risk modified by the likelihood of dying of other causes.

Examples of reductions in suicide rates in times of war have been described for several time periods and cultures [4-9]. Marshall [10] argued that the reduction in suicide rates reflected a reduction in unemployment, a recognised risk factor for suicide [11], rather than a direct effect of war, an argument offered some support by Lester and Yang [12]. Stack [13] suggested that the suicide rate decreased as the proportion of the population employed in military roles increased.

Work on recent conflicts has suggested a more complex effect. The war in Serbia and Croatia has been studied in some detail. Grubisic-Ilic et al [14] reported a decrease in suicide rates in parts of Croatia affected by war, while Bosnar et al [15] describe an increase in suicide rates in southern part of Croatia during wartime, with a particular increase in people aged under 40 years [16]. Grubisis-Ilic et al [14] and Bosnar et al [15,16] both report an increase in firearms deaths.

The last war with major involvement of the UK population was World War II. In the light of these conflicting findings we set out to examine suicide rates in Scotland, before, during and after the Second World War.

## Methods

The General Registrar Office for Scotland (GROS) provided information in the form of photocopies of the relevant data tables from GROS Annual Reports for the years 1931–1952. These Reports include all deaths within Scotland. Deaths among Scottish soldiers serving overseas during this time were recorded in separate records.

Three versions of the 'International Classification of Causes of Death' (ICD) were used by the GROS during the period 1931 to 1952 – ICD 4 during the years 1931 to 1940, ICD 5 during the period 1941 to 1949 and ICD 6 during the years 1950 to 1952. Information regarding the method of suicide is presented using the major categories outlined within ICD. The number of suicide deaths were extracted from the Annual Reports and entered onto computer spreadsheets. These were combined with population figures provided by the GROS to calculate suicide rates for all ages by gender, by gender and age group and by method and gender. The period 1939 – 1945 was classified as 'War' years. The periods 1931 – 1938 and 1946 – 1952 were categorized as 'non-War' years.

Forward stepwise logistic regression was used to assess the effect of gender, war and year on suicide rates. Three models were constructed for different hypotheses. Goodness of fit was tested by visual inspection of residuals. Data are presented as odds ratios with 95% Wald confidence intervals. SAS V8.2 was used for the analysis. Only effects whose inclusion was justified within the stepwise regression based on the chi-squared statistic are reported.

## Results

**Figure 1:**
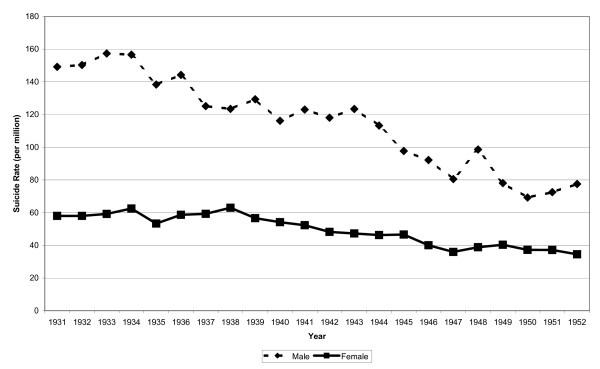
Suicide rate for all ages 1931 – 1952 in Scotland.

During the period 1931 – 1952, the overall suicide rate for both men and women declined (Figure 1) with a year-on-year Odds Ratio of 0.966 (0.962 – 0.969) as shown in Table 1. Among men, it fell from 149 per million in 1931 (347 deaths) to 78 per million (189 deaths) by 1952. The equivalent figures for women were 58 per million (146 deaths) and 35 per million (92 deaths) respectively. However, compared to both the pre-War and post-War periods, there was an increased likelihood of suicide among both men and women during the War years (Odds Ratio 1.085; 1.036 – 1.137), taking into account the fall in the rate during the War as part of a longer term trend. As shown in Table 1, men were significantly more likely to take their lives than women (Odds Ratio 2.328; 2.225 – 2.434) although there was no evidence of a statistically significant War × Gender interaction effect.

**Figure 2:**
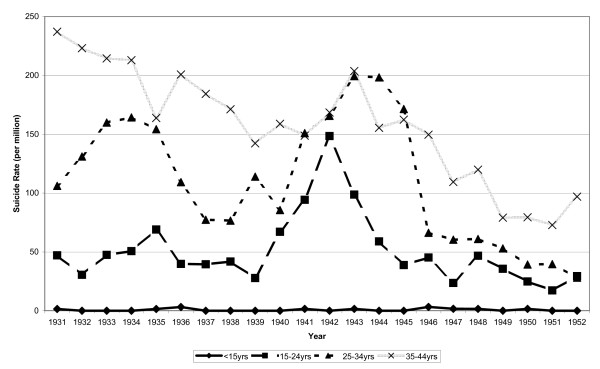
Suicide rate among men under 45 years in Scotland – 1931 – 1952.

**Figure 3:**
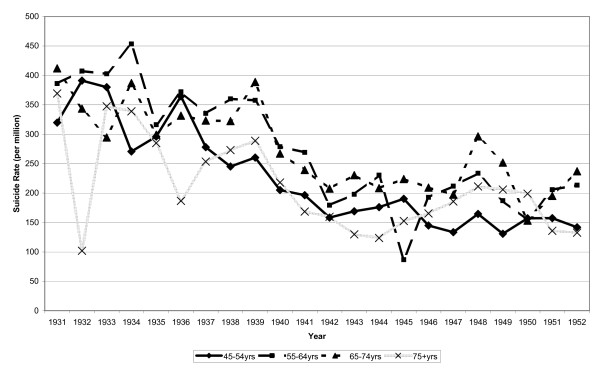
Suicide rate among men over 45 years in Scotland – 1931 – 1952.

There was variation in the suicide rate among different age groups. After the general trend for the whole period was modeled for, suicide rates among men aged 15 – 34 years rose during the period of the Second World War before declining to pre-war levels during the late 1940's (Figure 2). As shown in Table 1, this increase in the likelihood of suicide among men aged 15 – 34 years attributed to the War period was statistically significant (Odds Ratio 1.179; 1.1123 – 1.2492). Among men aged 15 – 24 years, the suicide rate peaked at 148 per million (41 deaths) during 1942 before declining to 39 per million (10 deaths) by 1945. The suicide rate among men aged 25–34 years reached 199 per million (43 deaths) in 1943, before falling to 66 per million (23 deaths) by 1946. A similar, but less striking, trend was seen among men aged 35 – 44 years, with rates rising and falling in the same period. The decline in suicide rates observed among men aged 45 years and over throughout the 1930's largely continued during the 1940's (Figure 3).

**Figure 4:**
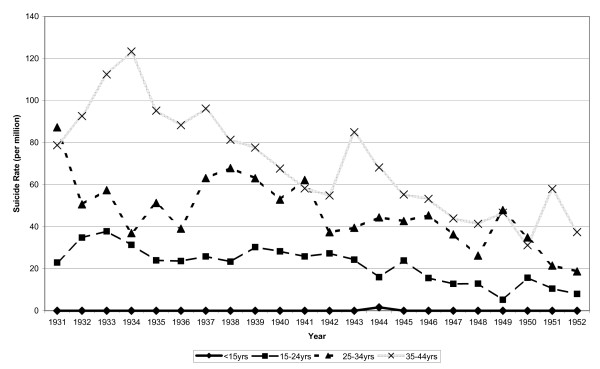
Suicide rate among women under 45 years in Scotland – 1931 – 1952.

**Figure 5:**
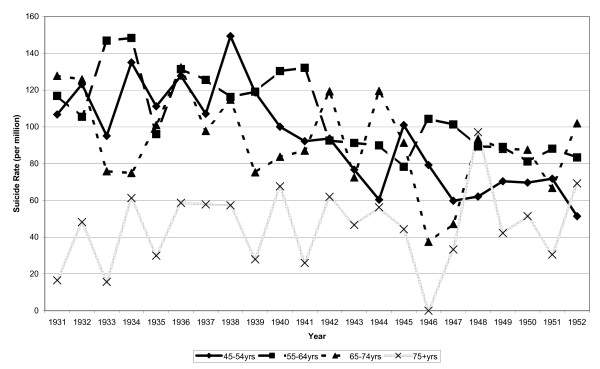
Suicide rate among women over 45 years in Scotland – 1931 – 1952.

Figures 4 and 5 show the suicide rate among women aged less than 45 years and 45 years and over respectively. Female age specific trends did not alter during the War years, with the exception of women aged 35 – 44 years. Suicide rates among these women rose during 1943, reaching 85 per million (33 deaths), before declining to 55 per million (22 deaths) by 1945.

**Figure 6:**
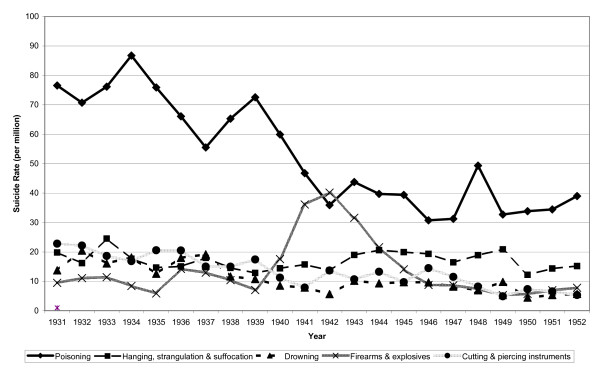
Suicide rate among men (all ages) by principal method in Scotland – 1931 – 1952.

**Figure 7:**
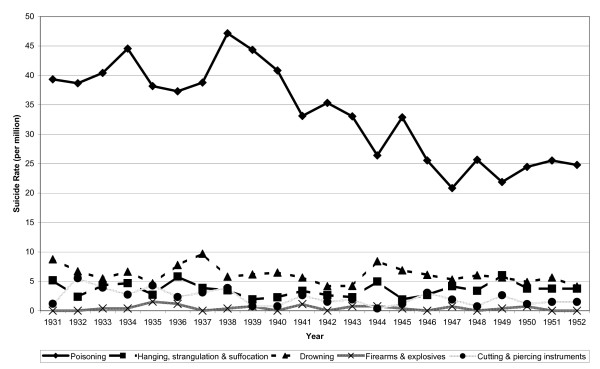
Suicide rate among women (all ages) by principal method in Scotland – 1931 – 1952.

Figure 6 shows the all age male suicide rate among men by principal method. Method specific trends evident during the 1930's were largely maintained throughout the 1940's, with the exception of suicides involving firearms and explosives. The suicide rate for this method rose markedly during the early 1940's, peaking at 40 per million (85 deaths) in 1942, before declining to 9 per million (20 deaths) by 1946. As shown in Table 1, this increase in suicides involving firearms and explosives associated with the War period was statistically significant (Odds Ratio 1.312; 1.210 – 1.422). There was no clear change to method specific trends among women during the period 1931 – 1952 (Figure 7).

## Discussion

Once the longer term trends are accounted for, suicide rates in younger men in Scotland rose during World War II, accompanied by an increase in suicide deaths in men involving firearms. Previous reports described a decrease in suicide rates in the UK in this period, but this related to total population figures [10] rather than to age-specific rates. Furthermore, in contrast to the established view on the effect of war on suicide rates, our results suggest that when compared to both the pre-War and post-War periods, the likelihood of suicide among the entire Scottish population during the Second World War was higher if the overall declining trend is taken into account.

Our study was limited to routine data. As we used collated figures, we were unable to cross tabulate age group, gender and method. The reliability of routine information is an important issue, but there was no clear reason why a death might be more likely to be recorded as suicide in wartime than in peacetime, so this seems an unlikely explanation. For the sake of parsimony we have treated the separate years as independent observations and have not attempted to estimate temporal auto-correlation. Future studies might examine this statistical question.

There are several possible explanations for these findings. Cultural attitudes towards suicide seem to be important [17] and these may change during wartime. One US study found that media coverage of suicide had less apparent impact on suicide rates in wartime than in peacetime, perhaps because of protective levels of social integration [18]. It may be, however, that social cohesion is felt more by non-combatant age groups than those in younger age groups. Suicide rates decreased among older men during the war, and the higher pre-war rates in these groups resulted in an overall decrease in the male suicide rate.

World War II, in contrast to more recent wars such as Korea and the Gulf Wars, exposed the home population to violence with bombing of UK targets. Strategic bombing has a large impact on civilian morale. As expected, the effects are greatest in those closest to the area being bombed, and this is partly mediated through disruption of day to day life [19]. In the recent Balkan war, suicide rates in people being treated for mental illnesses increased, as well as deaths in the general population [20]. There is no clear reason why social stresses would have a greater impact on younger men, and so other factors may be relevant.

The other main possibilities are the contribution of stressors in combatants, and of method availability. This study did not provide information on occupation, although the incidence of firearm deaths suggests that at least some deaths may have been in armed services or volunteer forces. Suicide in troops is well described [21], and firearms are often used. Increases in suicides involving firearms have been described in Serbia [22,23] and Croatia [15,16,24]. In a wider study in Croatia, Grubisic-Ilic et al [14] reported that firearms were used more in areas directly affected by war. This is relevant because of case-fatality rates. People using firearms to self-harm are very likely to die, compared to most other methods of self-harm. The relationship between firearm availability and suicide is well documented. This is not an exclusively North American phenomenon, and firearm ownership in some occupational groups is associated with higher rates of suicide [25-31].

It is of course possible that the increased likelihood of young males taking their lives during the early 1940's, and the increased likelihood of suicides involving firearms and explosives seen during this period, could reflect factors other than the War. However, given that analyses of suicide rates during recent conflicts have reported similar findings, it seems probable that the impact of the War was the major contributor.

## Conclusion

In contrast to the established view, our findings suggest that compared to both the pre-War and post-War periods there was an increased likelihood of suicide during the Second World War if the overall declining trend is taken into account. Furthermore, we conclude that the reduction in overall suicide rates in Scotland in World War II concealed an increase in rates in young men, and an increase in firearm deaths. Younger men are the groups most likely to be called to arms, and to have access to guns. It seems likely, therefore, that the stresses of being a potential combatant, and access to lethal methods of self-harm, were important contributors. This is supported by the minimal impact the war had on female suicide trends. Recent findings in central Europe were presaged by changes in Scotland more than half a century ago.

## Competing interests

The author(s) declare that they have no competing interests.

## Authors' contributions

RH contributed to the study design, obtained the data and created the spreadsheets. He contributed to the data analysis and writing of the paper. CS contributed to the study design, data analysis and writing of the paper. RWH and SS conducted the statistical analyses. All authors read and approved the final manuscript.

## Pre-publication history

The pre-publication history for this paper can be accessed here:



## Figures and Tables

**Table 1:** Results of logistic regression analysis

**Effect**	**Odds Ratio**	**95% CI**
**Is there any significant effect of war, gender and year on suicide rates?**		
Year	0.966	(0.962 – 0.969)
No War	1	
War	1.085	(1.036 – 1.137)
Female	1	
Male	2.328	(2.225 – 2.434)

**Is there higher suicide rate among young males (15–34 years)? If so, is it associated with war?**		
Year	0.961	(0.954 – 0.968)
No War	1	
War	1.141	(1.076 – 1.210)
Older	1	
Youth	0.793	(0.7479 – 0.8399)
War X Youth	1.179	(1.1123 – 1.2492)

**Was gunshot more prevalent of suicide amongst men during war time?**		
Year	0.963	(0.955 – 0.971)
War	1	
War	1.278	(1.178 – 1.386)
Method-Other	1	
Method-Gunshot	0.383	(0.353 – 0.415)
War W X Gunshot	1.312	(1.210 – 1.422)
